# Comparison of SMS-EPI and 3D-EPI at 7T in an fMRI localizer study with matched spatiotemporal resolution and homogenized excitation profiles

**DOI:** 10.1371/journal.pone.0225286

**Published:** 2019-11-21

**Authors:** Caroline Le Ster, Antonio Moreno, Franck Mauconduit, Vincent Gras, Ruediger Stirnberg, Benedikt A. Poser, Alexandre Vignaud, Evelyn Eger, Stanislas Dehaene, Florent Meyniel, Nicolas Boulant

**Affiliations:** 1 NeuroSpin, CEA, Université Paris-Saclay, Gif-Sur-Yvette, France; 2 NeuroSpin, CEA, Université Paris-Saclay, INSERM, Gif-Sur-Yvette, France; 3 German center for neurodegenerative diseases (DZNE), Bonn, Germany; 4 Department of Cognitive Neuroscience, Faculty of Psychology and Neuroscience, Maastricht University, Maastricht, The Netherlands; 5 Collège de France, Paris, France; Brigham and Women’s Faulkner Hospital, UNITED STATES

## Abstract

The simultaneous multi-slice EPI (SMS-EPI, a.k.a. MB-EPI) sequence has met immense popularity recently in functional neuroimaging. A still less common alternative is the use of 3D-EPI, which offers similar acceleration capabilities. The aim of this work was to compare the SMS-EPI and the 3D-EPI sequences in terms of sampling strategies for the detection of task-evoked activations at 7T using detection theory. To this end, the spatial and temporal resolutions of the sequences were matched (1.6 mm isotropic resolution, TR = 1200 ms) and their excitation profiles were homogenized by means of calibration-free parallel-transmission (Universal Pulses). We used a fast-event “localizer” paradigm of 5:20 min in order to probe sensorimotor functions (visual, auditory and motor tasks) as well as higher level functions (language comprehension, mental calculation), where results from a previous large-scale study at 3T (N = 81) served as ground-truth reference for the brain areas implicated in each cognitive function. In the current study, ten subjects were scanned while their activation maps were generated for each cognitive function with the GLM analysis. The SMS-EPI and 3D-EPI sequences were compared in terms of raw tSNR, t-score testing for the mean signal, activation strength and accuracy of the robust sensorimotor functions. To this end, the sensitivity and specificity of these contrasts were computed by comparing their activation maps to the reference brain areas obtained in the 3T study. Estimated flip angle distributions in the brain reported a normalized root mean square deviation from the target value below 10% for both sequences. The analysis of the t-score testing for the mean signal revealed temporal noise correlations, suggesting the use of this metric instead of the traditional tSNR for testing fMRI sequences. The SMS-EPI and 3D-EPI thereby yielded similar performance from a detection theory perspective.

## Introduction

With ultra-high fields (UHF), functional MRI (fMRI) benefits from increased signal-to-noise ratio (SNR) and sensitivity to BOLD contrast so that higher spatial resolutions can be reached. However, to maintain high temporal sampling rates for high spatial resolution, whole-brain coverage, the use of fast k-space sampling strategies becomes mandatory. Simultaneous multi-slice EPI (SMS-EPI) [1, 2] has marked a paradigm shift in functional neuroimaging [3]. The concurrent signal of multiple slices simultaneously excited by a multiband pulse is acquired in one shot, and with little SNR penalty that arises solely from the reconstruction process (g-factor noise). A key feature that enables the high slice acceleration therefore is the introduction of blipped-CAIPI (controlled aliasing in parallel imaging) gradient encoding along the slice direction [4] which leads to significant reduction in g-factor penalty by effectively maximizing the distance between aliased voxels [5]. The main challenge yet with the SMS-EPI approach is the inherent high energy deposition in the tissues engendered by shorter repetition times, especially at UHF, and peak power demands [2]. Alternatively, for similar applications, functional imaging can be performed with a 3D-EPI sequence with significantly lower flip angles [6]. Here, the acquisition of the 3D k-space is segmented into multiple shots. Following each slab- or spatially non-selective excitation, an EPI kz-space plane is acquired and prepared with a secondary phase encoding step along the slice direction (a.k.a. partition). Since there are now two phase encoding directions, each can be undersampled so that 3D EPI offers the same acceleration capability as SMS-EPI. Moreover, the 3D k-space sampling can also be combined with 2D-CAIPIRINHA [5] to decrease geometry-dependent noise penalty [7]. In conjunction with sagittal slice placement, the use of non-selective pulses results in minimal energy deposition, especially when employing relatively long pulses for water-selective excitation [8]. A potential drawback of 3D-EPI on the other hand is its greater sensitivity to physiological noise [9] which may adversely affect temporal SNR (tSNR) and hence functional sensitivity. Recent studies at 3T [10, 11] and 7T [12–14] however have shown that proper modelling of the physiological noise [15] can improve the tSNR of 3D-EPI so as to outperform SMS-EPI acquisitions. This is particularly the case when the baseline SNR is sufficiently high to be in a physiological noise-dominated regime [16], i.e. when dealing with low to moderate spatial resolutions. At high spatial resolution, 3D-EPI in principle outperforms SMS-EPI acquisitions because of the higher intrinsic SNR according to the theory [6].

Despite the dominating benefit of superior tSNR and BOLD sensitivity at UHF, a considerable impediment is the transmit B1^+^ field inhomogeneity. This can result in local signal voids, impairing signal and hence tSNR most dominantly in the temporal lobes and cerebellum. In addition, the specific flip angle excitation patterns of the SMS-EPI and 3D-EPI sequences result in different signal responses that play a role in the comparison of these sequences, especially at 7T. Several approaches have been proposed to achieve homogeneous excitation flip angles across the whole volumes, such as dielectric pads [17] and parallel transmission (pTx) [18, 19]. In pTx, an array of local transmit antennas (typically 8) is used to deliver radiofrequency (RF) pulses, allowing the RF amplitude and phase waveforms on each channel to be varied independently so as to return an overall more homogenous excitation. This is achieved by stepping through a given k-space trajectory during excitation to yield either slice-selective excitation (spokes) [20, 21] or nonselective excitation patterns (kT-points) [22]. For a recent review on the topic of pTx, the reader is referred to [23]. The downside of pTx which also has so far hindered its widespread routine application is the need for a typically lengthy subject-specific precalibration protocol, comprising B1^+^ mapping, static field (ΔB0) mapping, brain masking and on-the-fly RF pulse design to calculate the optimal RF and gradient waveforms. The concept of universal pulses (UP) was recently introduced in order to skip this time-consuming step and simplify the acquisition workflow [24, 25]. UPs exploit the similarities of B1^+^ and ΔB0 over subjects: using a database of B1^+^ and ΔB0 maps from a collection of subjects, the pulses are designed to achieve the best possible flip angle homogeneity over this representative population for given hardware and SAR constraints. The resulting UPs can then easily be applied on any subject without the need for subject-specific calibration scan or pTx-related operator interactions. Following previous demonstrations of UPs in anatomical imaging sequences (SPACE, MPRAGE, FLAIR, DIR) and their robustness across multiple sites [26], the concept was recently extended to SMS-EPI [27] and 3D-EPI sequences [28] to bring their benefit to whole-brain functional MRI acquisitions as well in the context of resting-state fMRI. For both cases, superior performance was observed with UP compared to single channel excitations.

The aim of the current study hence was to compare the performance of the SMS-EPI and the 3D-EPI sequences in detecting a range of task-evoked activations across different brain areas, with similar homogenized excitation profiles by using UPs. Metrics such as tSNR [29] and functional contrast to noise ratio, while very useful, ignore temporally stable artefacts (e.g. from calibration, ghosts, slice leakage etc) and can confound neural-induced signal changes as noise. Finally, recent results have shown that t-score results could be amplified if temporal correlations in the noise were not properly modelled, suggesting the more relevant metric of t-score testing for the mean signal for benchmarking fMRI sequences and protocols [30, 31]. To this end, here a robust fMRI localizer paradigm was applied to 10 healthy adult volunteers at 7T as an attempt to evaluate two state of the art sequences with a real-world fMRI application. The two sequences were matched for spatial and temporal resolutions (TR = 1200 s, 1.6 mm isotropic voxels). The functional localizer paradigm used in the current study probes various cognitive functions, from sensorimotor (visual and auditory perception, motor actions) to higher levels (language comprehension, mental calculation), and thus includes some regions that are particularly prone to B1^+^ deviations. The brain regions implicated in each of the sensorimotor functions have been well characterized in previous work on N = 81 subjects at 3T, where robust and reliable activation across subjects was observed with a 5 min paradigm [32], with little intra/inter-subject variability. This battery of tasks thus provides a test bed for evaluating and comparing different fMRI acquisition schemes. Given their high-reproducibility across subjects and sessions, the functional brain maps identified in the large-scale study here served as the ground truth for benchmarking the two sequences by comparing their ability to detect the brain regions implicated in different sensorimotor functions. We quantified detection performance in terms of sensitivity and specificity, using standard task fMRI analysis and tools from detection theory [33, 34], and put the results in perspective with raw tSNR, t-score testing for the mean signal and t-score metrics.

## Materials and methods

### MR imaging

This study was approved by the local Institutional Review Board (Comité de protection des personnes Sud Méditerranée, approval number 2018-A01761-54) and the volunteers provided informed written consent. Acquisitions were performed on 10 healthy volunteers (23±3 years, 5 females) on a Magnetom 7T scanner (Siemens Healthineers, Erlangen, Germany) equipped with the Nova (Nova Medical, Wilmington, MA, USA) 8Tx-32Rx head coil. For each volunteer, a second order shim was performed, then an anatomical UP-enabled MPRAGE sequence [25] was acquired, followed by the SMS-EPI and 3D-EPI functional sequences acquired in a pseudo-randomized order with whole-brain coverage. The acoustic noise level of the final measurement protocols was measured prior to *in-vivo* exams with a sound pressure meter placed at a location equivalent to the right ear of the volunteers. In a pilot study, physiological data (cardiac pulsation and respiration) were recorded during the functional scans. As their inclusion in the analysis did not improve the detection of activation ROIs, they were not subsequently recorded.

Imaging parameters of the UP-MPRAGE sequence were: TR/TE/TI 2600/3.4/1100 ms, flip angle (FA) 4°, 0.8 mm isotropic resolution, 192 sagittal slices, FOV 256 mm, parallel imaging with GRAPPA (R = 2), resulting in an acquisition time of 6:35 min. Besides a marginally longer TR, parameters of the SMS-EPI sequence closely matched the ones of the 7T Human Connectome Project (HCP) resting-state fMRI protocol [35]: TR/TE 1200/22 ms, 1.6 mm isotropic resolution, 90 axial slices, FOV 208 mm, PF = 7/8 along the phase encoding direction, parallel imaging with GRAPPA R = 2, BW = 2024 Hz/pixel, MB factor = 5, FA = 55°, fat saturation, CAIPI shift = FOV/3, anteroposterior phase encoding, echo spacing = 0.6 ms. Online image reconstruction was performed with the implementation of the MGH blipped-CAIPI MB-EPI C2P (www.nmr.mgh.harvard.edu/software/c2p/sms), which uses sequential application of Slice-GRAPPA [4] with leak-block [36] and GRAPPA [37]. The same parameters were used for the 3D-EPI sequence except for R = 2×4 along the phase and partition encoding directions respectively (CAIPI shift Δk_z_ = 2), sagittal slice orientation, FA = 12° and no fat saturation owing to spatially non-selective water excitation [8]. Reconstruction was carried out using the 2D Caipirinha GRAPPA algorithm (kernel size 3×4) provided by the vendor. The respective target FAs were chosen to be equal to the Ernst angle for grey matter (T1 = 2000 ms). For each sequence, 5 additional volumes were acquired with reversed phase-encoding to perform subsequent distortion correction.

### Pulse design

Universal Pulses were designed for the SMS-EPI and the 3D-EPI sequences using the offline flip angle (FA) normalized root mean square error (NRMSE) minimization algorithm described in [24]. The average NRMSE was minimized over a database comprising brain-masked B1^+^ and ΔB0 field maps of 20 subjects that were collected in a previous study [25]. The pulse design was performed under hardware and SAR constraints using virtual observation points (VOPs) [38] in compliance with the International Electrotechnical Commission guidelines in the normal mode of operation, i.e. with 3.2 W/kg global SAR and 10 W/kg local SAR limits. Average power limits at the coil plug were set by the coil manufacturer as 3 W per channel and 16 W total. The limit in peak amplitude was set to 170 V. The VOPs were made and validated in house using electromagnetic simulations provided by the coil manufacturer. They incorporated modelling errors, intersubject variability and directional coupler uncertainty safety factors to cumulate a 2.3 safety margin [39].

A waveform with two spokes for each slice was chosen for the design of the UP of the SMS-EPI sequence [27]. The duration of each sub-pulse was set to 2.2 ms (time-bandwidth product = 3.2), with a composite RF spacing of 2.4 ms and a bipolar scheme corrected with phase offsets to compensate for gradient delays [40]. For simplicity, peak power for the multiband approach was handled in the algorithm by assuming constructive interference between the waveforms constituting a same group. For each set generated, FA maps were simulated using Bloch equations for all the subjects of the database. For the 3D-EPI sequence, the UP was designed as a non-selective kT-point RF pulse with 3 square sub-pulses of 1 ms long each, in order to be water-selective without extra need for fat saturation [8]. This number of kT-points was chosen as a compromise between RF field inhomogeneity mitigation and total pulse duration to allow matching the desired TR while being water-selective. For both sequences, the complex weights and k-space locations of each channel and each kT-point were concurrently optimized [41]. Due to the non-convexity of the problem, the optimization was repeated with multiple k-space initializations and the best outcome was kept for implementation. Obtained pulses were then applied on the 10 volunteers of the current study with neither pTx calibrations nor additional calculations. During the exam, the sequences were run under local SAR supervision with the home-made VOPs.

### Paradigm

**Fig 1:**
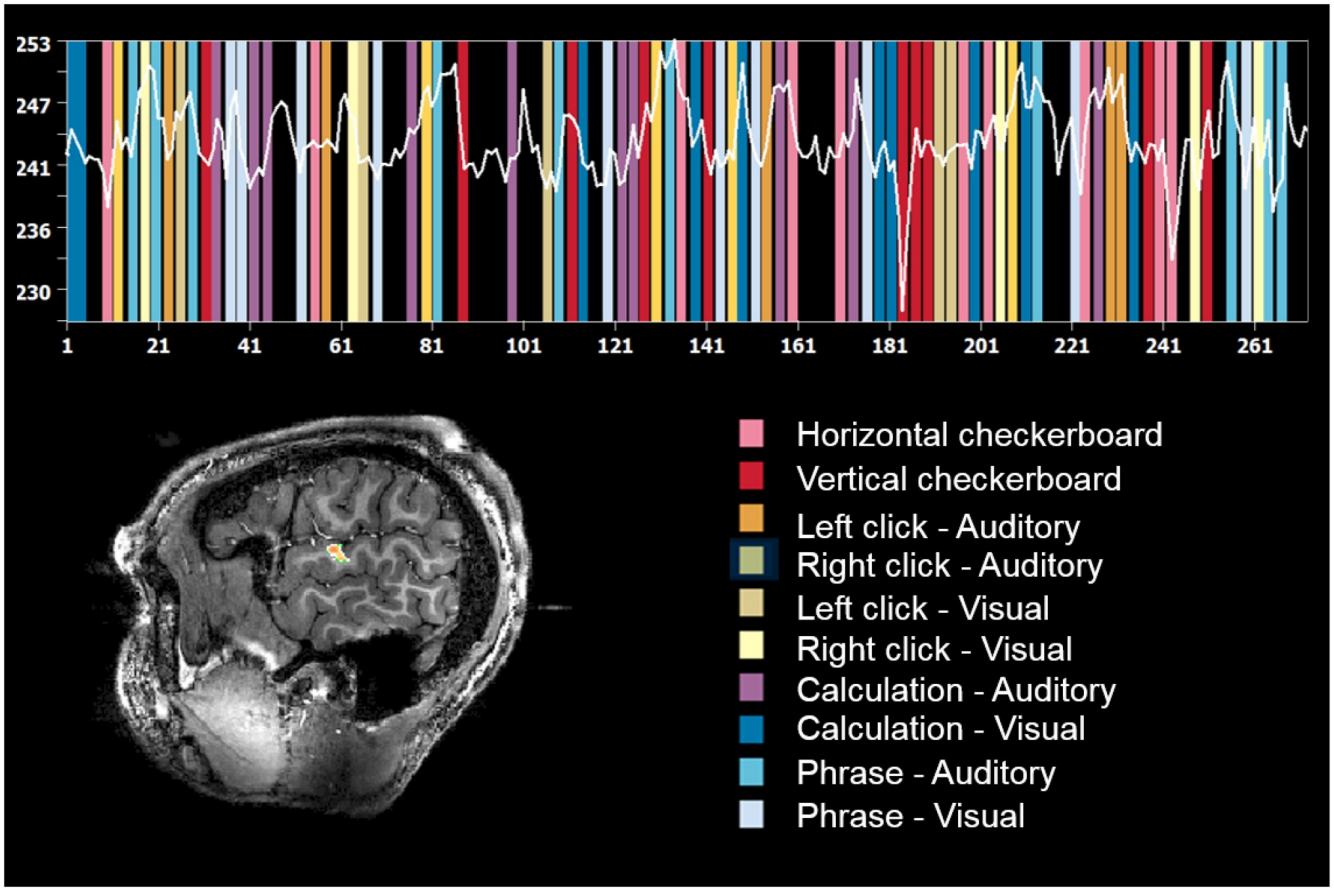
Paradigm description. Illustration of the succession of tasks occurring during the localizer with an example of signal recorded in a ROI of the temporal lobe of one volunteer.

The functional localizer consisted of a fast event-related paradigm of 5:20 min duration as previously applied in the 3T study introduced above and used as reference [32]. It was also used as benchmark for comprehensive evaluation of increasing temporal resolution with multi-band accelerated protocols [42]. It elicits task activation in different sensorimotor areas (visual and auditory perception, motor action) and associative areas (mental calculation, language comprehension). This localizer includes ten different types of tasks, as shown in Fig 1. The sentences and calculations were varied each time. A total of four different localizers were designed with varying sentences and computations. For each volunteer, a different localizer was used for the SMS-EPI and 3D-EPI sequences in order to minimize habituation effects. The choice of the localizer was pseudo-randomized across volunteers. Stimulus design and delivery were performed with Python.

### Data analysis

#### Post-processing pipeline

Data were preprocessed with SPM12 (R7219, http://www.fil.ion.ucl.ac.uk/spm) [43] for all steps, apart from distortion correction with Topup [44] and tissue segmentation with FAST [45] which are part of the FSL library (FMRIB, Oxford, UK). The SMS-EPI data were first slice-time corrected, then both the SMS-EPI and 3D-EPI time series were realigned for rigid body motion (6 d.o.f.), and finally distortion corrected. For each subject, the T1-weighted anatomical scan was segmented into grey matter, white matter and cerebrospinal fluid, bias corrected and spatially normalized to the 2 mm brain template of the Montreal Neurological Institute (MNI152), and the same transformations were applied to the preprocessed functional volumes projected to the anatomical scans. Then, functional volumes were smoothed with a 5 mm (full-width at half maximum) Gaussian filter. Individual time series of the voxels were also high-pass filtered with a cut-off period of 128 s to remove slow signal drifts, and whitened using two different models for comparison purposes: the first-order auto-regressive model, referred to as AR(1), and the FAST model implemented in SPM which is a more complex model that accounts for high order temporal correlations in the noise [31, 46]. The analysis was restricted to a thick band around the grey matter, to account for smoothing, defined here as voxels with a probability of pertaining to the grey matter of at least 5% in SPM’s template tissue probability map.

Univariate activation maps were obtained by fitting onto a general linear model (GLM). First, we only included the temporal onsets of each stimulus convolved with the canonical hemodynamic response function (default parameters in SPM) and their first derivative in order to account for latency in BOLD response. We calculated linear contrasts of parameter estimates to test the effect of each task (e.g. right vs. left button press) and tested their significance with mass-univariate (voxel-wise) t-tests. A p-value of 0.001, uncorrected for multiple comparisons, was chosen as the significance threshold since it is the threshold used for subject-level analysis in the study that we used as a reference [32]. The mild impact of the physiological regressors from the respiratory and cardiac recordings observed in the pilot study may have been due to a poor quality of the recordings. As a result, we performed instead with nilearn (https://nilearn.github.io/) a CompCor [47] analysis to return 3 cerebrospinal fluid and 5 white matter related physiological regressors. These regressors were derived for each subject from the cerebrospinal fluid and white matter masks obtained from the probability maps returned by the segmentation algorithm for a threshold of probability equal to 1. Data analysis was performed with and without inclusion of the nuisance (i.e. movement and physiological) regressors in the GLM. In each case, SPM returned t-score maps which were taken as indicative measures of activation strength.

#### Comparison strategy

The SMS-EPI and 3D-EPI sequences were compared in terms of raw tSNR, t-score testing for the mean signal [31], activation strength and activation accuracy. tSNR maps were computed for each subject as the mean over the standard deviation of the raw time-series of the functional scans for each voxel after realignment, distortion correction, detrending and normalization on the 2 mm resolution MNI brain template. tSNR maps were then averaged over the population. The t-score testing for the mean signal was simply returned by SPM with the contrast vector corresponding to the mean signal regressor. It was thus computed from the post-processed images normalized on the 2 mm resolution MNI brain template and averaged over the population.

Activation strength maps were computed by averaging the t-score maps for each contrast over the population. The contrasts were the following: video—audio (all visual stimuli versus all auditory stimuli), audio—video, left button press—right button press (button press conditions both visual and auditory, left versus right), right button press—left button press, video sentences—checkerboard (all video sentences versus blinking checkerboard) and computation—sentences (visual and auditory computation conditions versus visual and auditory sentences).

In this context, the SMS-EPI and 3D-EPI sequences were evaluated according to their capability to correctly detect activation, in comparison with the reference activation maps reported at 3T [32], for the same linear contrasts. Those maps were computed by using a random effect analysis and shall be denoted here as ROI_ref_. The four sensorimotor contrasts that were shown to be robust versus inter-subject and inter-session variability were used for benchmarking. On the other hand, the higher level networks (language comprehension, mental calculation), though activated, were not used for this quantitative benchmark because of considerably higher intersubject variability [32]. A good fMRI sequence should maximize the detection of actual activations (i.e. have a good sensitivity) while minimizing the detection of false activations (i.e. have a good specificity). Sensitivity corresponds to the rate of true positives; in this study it was computed for each of the four sensorimotor contrasts as the number of activated voxels in ROI_ref_ over the total number of voxels in ROI_ref_. Specificity corresponds to 1—the rate of false positives; it was computed for each of the four sensorimotor contrasts as 1—the number of activated voxels outside ROI_ref_ over the total number of voxels outside ROI_ref_. Note that both sensitivity and specificity depend on the significance threshold used to define activated vs. inactivated voxels. The sensitivities and specificities of the SMS-EPI and 3D-EPI sequences were computed for significance thresholds ranging from 0 to 0.5 to build the Receiver Operating Characteristic (ROC) curve [33]. Their relationship was then plotted by averaging the sensitivity and specificity obtained for the four sensorimotor contrasts over the 10 subjects. From this curve, the area under the curve (AUC) can be computed to measure the discriminating power of the sequence: it is equal to 1 in case of a perfect classification and 0.5 for a random classification. The AUC was computed for the SMS-EPI and 3D-EPI sequences and averaged over the 10 subjects. In addition, following detection theory [34], the d’ scores of these sequences were computed for p<0.001, uncorrected, as a measure of the distance between the normalized true positive and false positive rates, which should be maximized: d’ = Z(sensitivity)—Z(1—specificity), Z being the inverse of the cumulative normal distribution function. When the distributions of those rates are Gaussian with equal variance, d’ is a constant (independent from the significance threshold used) related to the AUC. Often in practice, these assumptions can be violated and d’ depends on the significance threshold. The AUC therefore summarizes the discriminating power over a range of p-values while the d’ provides the same kind of performance metric but for a particular p-value. Unless otherwise specified, the results presented in the following section were obtained from the GLM analysis performed with inclusion of the nuisance regressors and FAST noise whitening model.

## Results

**Fig 2:**
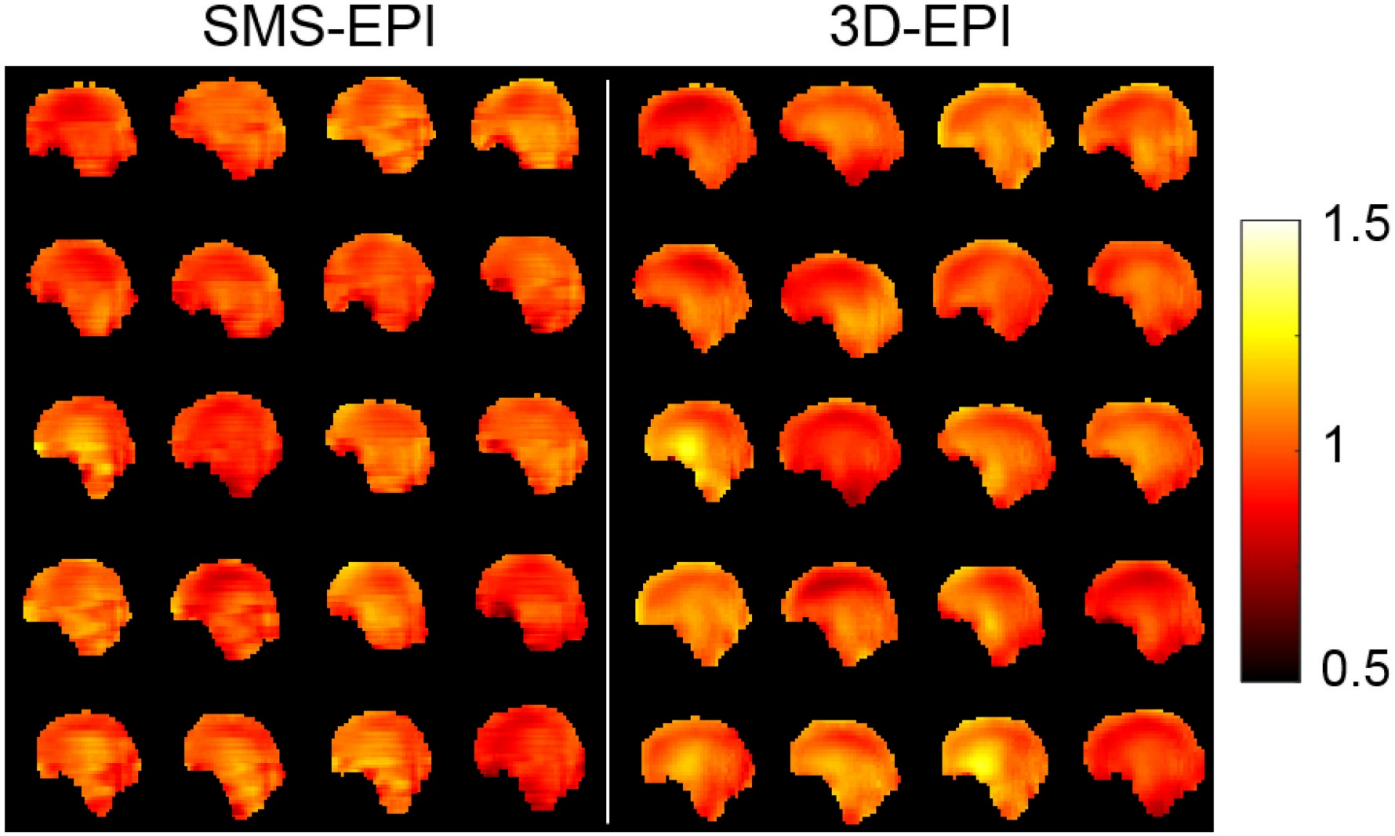
Normalized flip angle maps. Results of the simulations obtained with universal pulses over the 20 subjects of the pulse design database for the SMS-EPI (left panel) and 3D-EPI (right panel) sequences, in the mid-sagittal plane.

**Fig 3:**
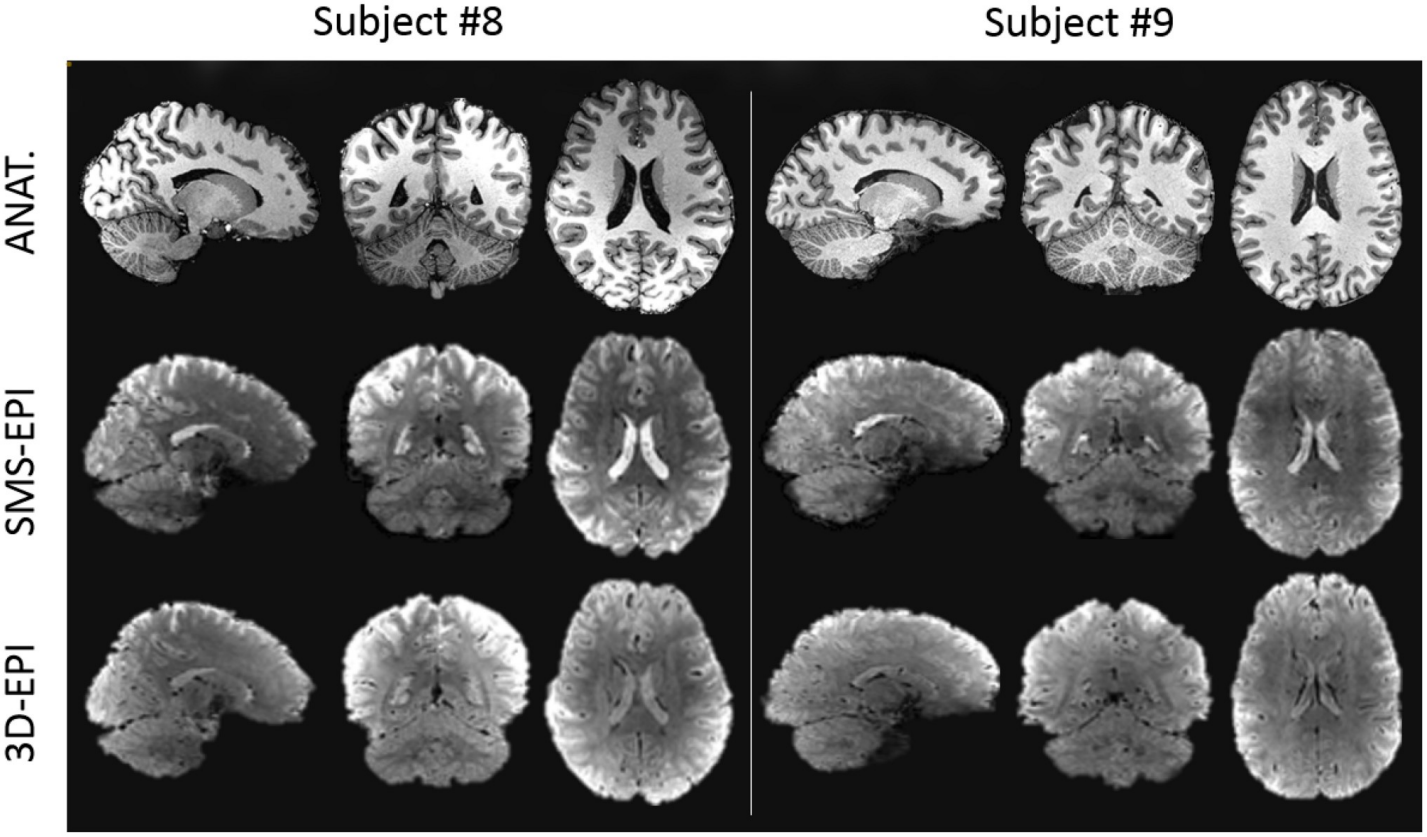
Example of *in-vivo* anatomical and EPI raw images. Example of images acquired on two subjects in the sagittal, axial and coronal planes. The first row represents the anatomical scan (MPRAGE), while the second and the third rows represent the functional scans acquired with the SMS-EPI and 3D-EPI sequences, respectively. The functional images displayed were realigned and corrected for distortion.

Normalized flip angle maps simulated with Bloch equations over the 20 subjects of the pulse design database are provided in Fig 2. Estimated flip angle distributions in the brain reported a normalized root mean square deviation from the target value of 7.4% for the SMS-EPI sequence and 8.1% for the 3D-EPI sequence, on average over the subjects. The returned peak 10-g SAR was 9.9 W/kg for the SMS-EPI sequence and 0.2 W/kg for the 3D-EPI sequence, saturating in the former case the normal mode IEC guidelines with the currently enforced safety margins. The global SAR was 1.79 W/kg for the SMS-EPI and 0.03 W/kg for the 3D-EPI. Average sound pressure level was about 10 dB higher for the SMS-EPI sequence (114 dB) than for the 3D-EPI sequence (105 dB). Examples of *in-vivo* EPI raw images acquired over two subjects of the current study are provided in Fig 3.

**Fig 4:**
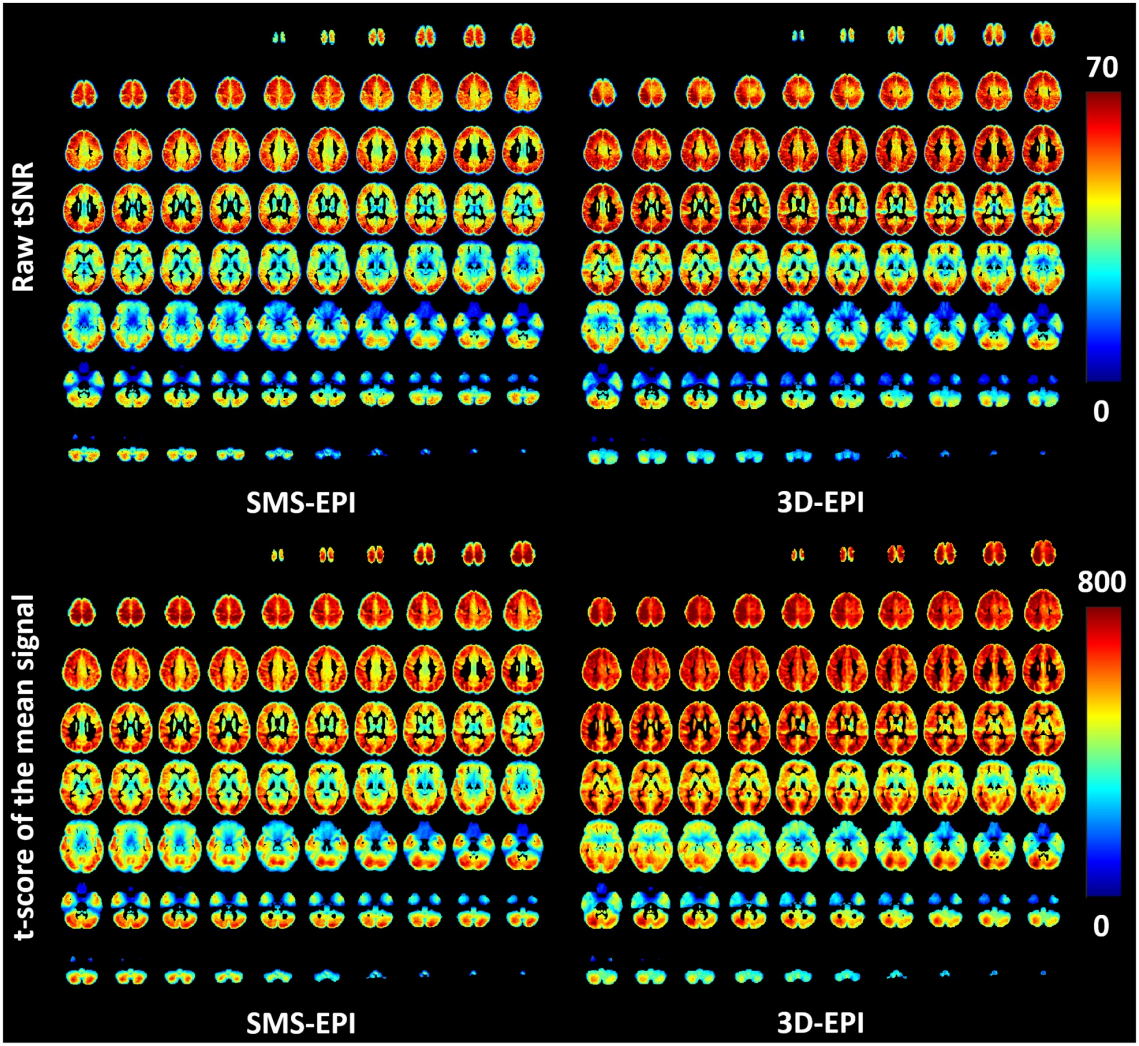
tSNR maps. Raw tSNR (upper row) and t-score testing with FAST for the mean signal (lower row) measured on average over the population for the SMS-EPI and 3D-EPI sequences.

The raw tSNR maps averaged over the subjects of the current study and normalized on the 2 mm isotropic MNI template are displayed in Fig 4. These maps show different tSNR patterns for the two sequences, with 41±17 versus 38±15 mean tSNR values for the 3D-EPI and SMS-EPI sequences respectively. In the same figure, the t-scores of the mean for the two sequences are also reported. On average, it was higher for the 3D-EPI sequence (544±157 versus 496±155), especially in the central parts of the brain.

**Fig 5:**
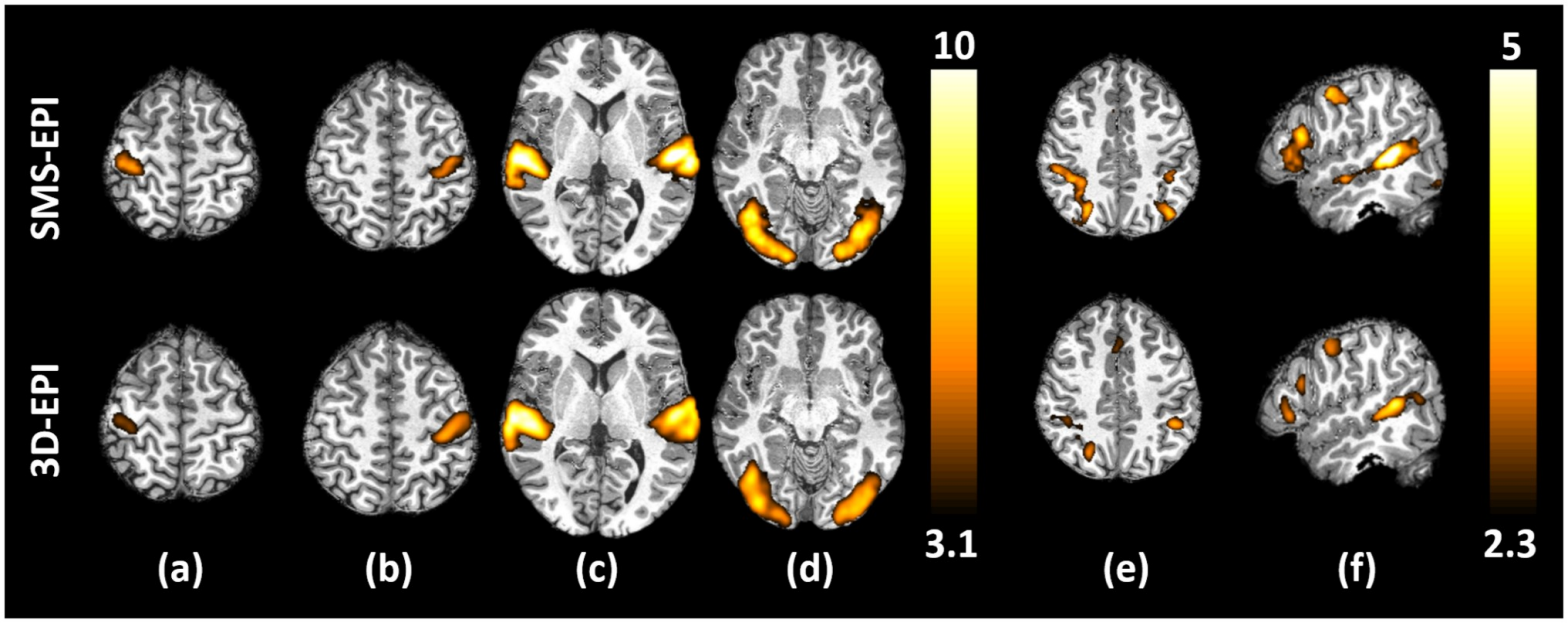
Activation maps. t-score maps averaged over the 10 volunteers for (a) the right>left click contrast, (b) the left>right click contrast, (c) the audio>video contrast, (d) the video>audio contrast, (e) the computation>sentences contrast and (f) the sentences>checkerboard contrast. The sensorimotor contrasts (a to d) were thresholded at p<0.001, uncorrected, while the higher level contrasts (e and f) were thresholded at p<0.01, uncorrected.

Brain areas activated by sensorimotor and higher level contrasts of the localizer and averaged over the subjects are displayed in Fig 5 for a significance threshold of p<0.001, uncorrected (sensorimotor contrasts) and p<0.010, uncorrected (higher level contrasts). As expected, the motor tasks activated the right and left central sulci, the auditory stimuli activated the temporal lobes, the visual stimuli activated the occipital lobes, the video sentences activated the language reading network (left superior temporal sulcus, inferior frontal gyrus, fusiform gyrus) and the mental calculations activated the computation network (parietal lobe). The t-scores were higher for the sensorimotor contrasts compared to the higher level contrasts due to both stronger activations and higher inter-subject reproducibility. In general, the activation strength of the SMS-EPI was slightly higher than the 3D-EPI sequence, especially for the higher level contrasts. The t-score maps displayed here being averaged over the population, they tend to hinder subject-specific false positive activations and thus can be seen as a qualitative estimate of the sequence sensitivity.

**Fig 6:**
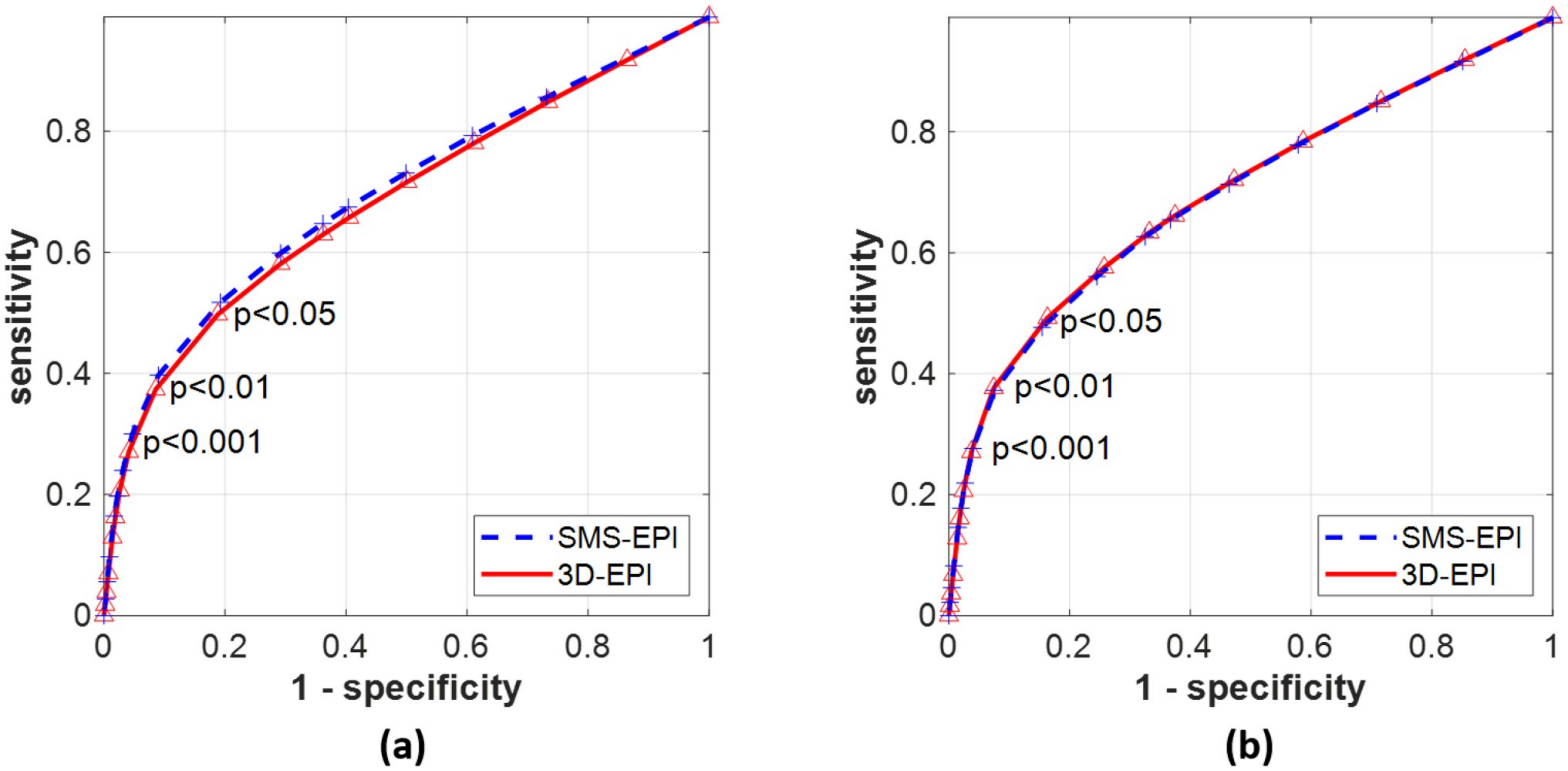
ROC curves. ROC curves obtained for the SMS-EPI (blue dashed line) and 3D-EPI sequences (red solid line) for the GLM analysis performed with AR(1) (a) and FAST (b) noise whitening models. These curves were obtained by averaging the mean sensitivity and specificity measured for the four robust sensorimotor contrasts over the subjects for significance thresholds ranging from 0 to 0.5.

**Fig 7:**
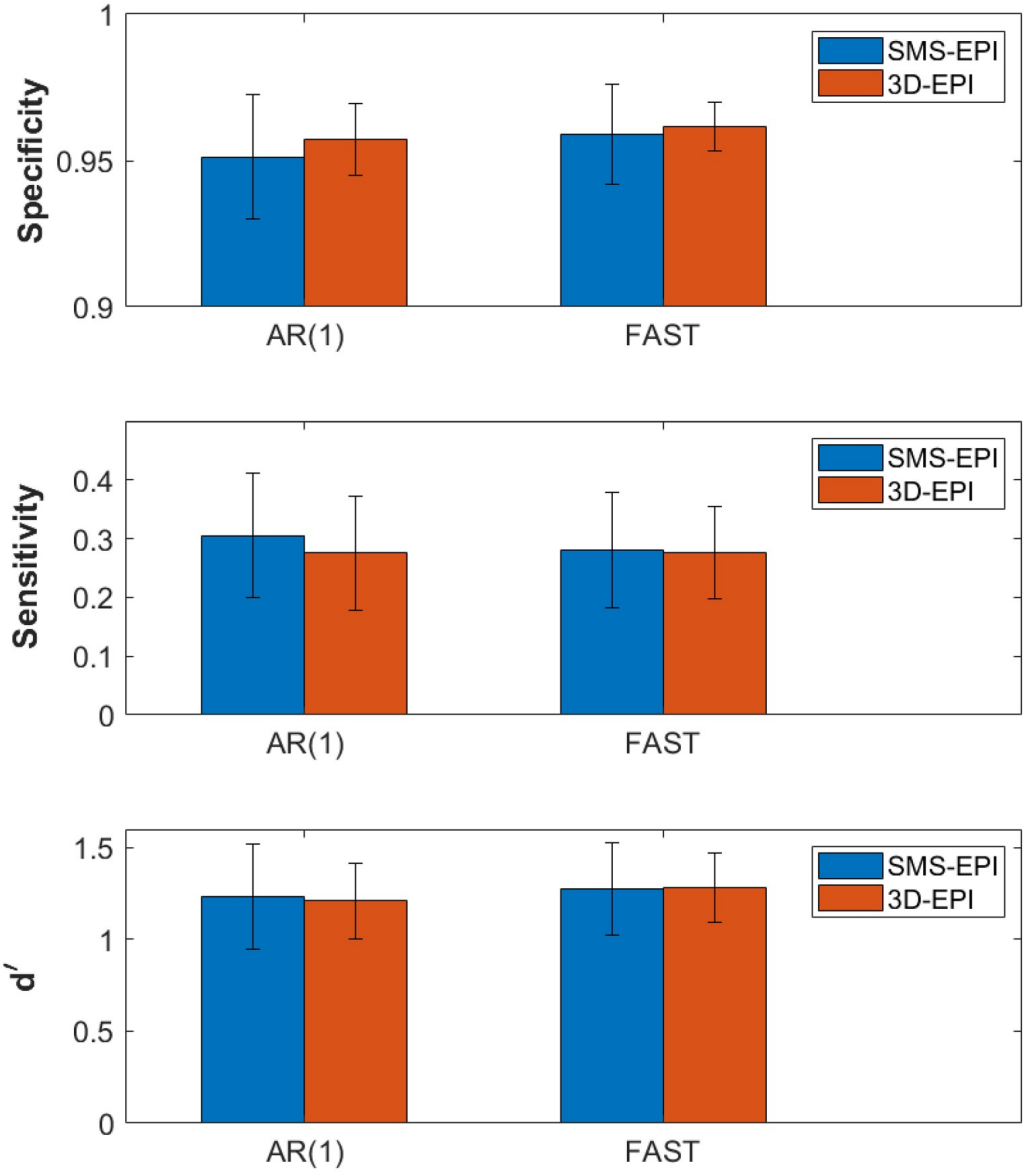
Activation accuracy. Bar-plots representing the specificity, sensitivity and d’ values for the SMS-EPI and 3D-EPI sequences, and computed with AR(1) and FAST noise whitening models. These values were measured by averaging the mean values obtained for the four sensorimotor constrasts over the subjects for a significance threshold p<0.001, uncorrected.

The ROC curves obtained by comparison of the activation maps to the reference ROIs and averaged over the four sensorimotor contrasts are displayed in Fig 6. The mean AUC±std of the ROC curves over the subjects were: AUC_SMS-EPI_ = 0.70±0.06 vs. AUC_3D-EPI_ = 0.68±0.06 for the analysis performed with AR(1); AUC_SMS-EPI_ = 0.70±0.05 vs. AUC_3D-EPI_ = 0.70±0.06 for the analysis performed with FAST. The mean sensitivity, specificity and d’ results obtained with AR(1) and FAST noise whitening models for p<0.001 are reported in Fig 7. The 3D-EPI showed a trend of lower sensitivity and higher specificity compared to the SMS-EPI sequence, these differences were however not statistically significant neither for AR(1) (paired t-test with 9 degrees of freedom, p = 0.58 and t = 0.57 for the sensitivity; p = 0.44 and t = 0.81 for the specificity) nor for FAST (p = 0.91 and t = 0.12 for the sensitivity; p = 0.68 and t = 0.43 for the specificity). In addition, noise whitening with FAST compared to AR(1) increased the specificity and lowered the sensitivity, especially for the SMS-EPI sequence (p = 0.01 and t = 0.01 for the sensitivity; p = 0.03 and t = 0.02 for the specificity) and to a lower extent for the 3D-EPI sequence (p = 0.99 and t = 0.01 for the sensitivity; p = 0.16 and t = 0.08 for the specificity). In the end, when combined, the two sequences returned similar performance from a detection theory perspective for both AR(1) and FAST noise whitening models: d’_SMS-EPI_ = 1.23±0.29 vs. d’_3D-EPI_ = 1.21±0.21 for AR(1) and d’_SMS-EPI_ = 1.28±0.25 vs. d’_3D-EPI_ = 1.28±0.19 for FAST. When the nuisance regressors were not included in the GLM analysis, these values read: d’_SMS-EPI_ = 1.20±0.29 vs. d’_3D-EPI_ = 1.12±0.23 for AR(1) and d’_SMS-EPI_ = 1.29±0.15 vs. d’_3D-EPI_ = 1.21±0.22 for FAST. The improvement of including the nuisance regressors in the GLM in terms of d’ was higher for the 3D-EPI (p = 0.06 and t = 2.17 for AR(1), p = 0.02 and t = 2.83 for FAST) compared to the SMS-EPI sequence (p = 0.47 and t = 0.76 for AR(1), p = 0.80 and t = 0.26 for FAST). For testing purposes, data processing was also performed with 0 and 2 mm smoothing kernels. Resulting d’ were lowered, especially for the 3D-EPI sequence: it was 36% and 11% lower without smoothing for the 3D-EPI and SMS-EPI sequences, respectively, while it was 8% and 1% lower with the 2 mm smoothing kernel for the 3D-EPI and SMS-EPI sequences, respectively. Reducing the smoothing kernel size particularly lowered the specificity of the 3D-EPI sequence.

## Discussion

In the current study, we showed that the performances of the SMS-EPI and 3D-EPI sequences to detect visual, auditory and motor contrasts were equivalent in a multi-task fMRI experiment performed at 7T and using pTx UPs, for matched spatial and temporal resolutions, highly similar to that of the resting-state fMRI protocol of HCP [48]. In this context the use of detection theory [33, 34] for benchmarking implicitly involved subject-level analysis, as opposed to group analysis. The sequences nevertheless showed different behaviors in terms of tSNR, t-score testing for the mean, sensitivity and specificity.

In the current study, the UPs allowed for straightforward and operator independent application to functional MRI, with neither time penalty for calibration scans (B1^+^), pulse calculation (tailored pulse design) or other pTx-related steps (e.g. brain masking for pulse optimization). A study focusing on the improvements brought by UPs versus single channel quadrature excitations for the SMS-EPI and for the HCP-style resting-state fMRI protocol was also reported in [27] with up to 2 fold local tSNR boost and 25% on average. A similar study was also performed for the 3D-EPI sequence [28] but with more moderate gains due to the increased robustness of the signal versus the flip angle around the Ernst angle for short TR sequences. Here both protocols were operated in such optimized pTx regimes owing to the universal pulses. As shown in previous work, little would be gained with subject-based tailored pTx approaches [24, 27]. As shown also in [49], head displacement throughout the exam causes changes in the RF field maps typically acquired at the beginning of the exam and thereby affects pulse performance. Universal pulses are by construction more immune to this phenomenon because they are more broadband and are designed on a database of field maps incorporating different head positions. tSNR gains with versus without B1^+^-inhomogeneity mitigation schemes obviously depend on the sequence details. In this study, not employing such schemes would clearly have an impact on the comparison results.

The SMS-EPI sequence saturated the peak 10-g SAR constraint, due to both the short TR with whole-brain coverage and the application of a relatively high flip angle. In comparison, the 3D-EPI sequence benefited from a much lower energy deposition due to the relatively long 3D water-selective excitation and a lower flip angle. Water selection furthermore allowed sparing a SAR and time-consuming fat saturation pulse, while enabling a lower partition acceleration factor of 4. Undersampling factors for the SMS-EPI and 3D-EPI sequences were therefore different. Although different acceleration factors definitely play an important role in the final SNR, the TR was considered the most crucial parameter to match in this neuroscientific context [11]. In case of partial brain coverage utilizing slab excitations in the 3D-EPI sequence, the RF pulses would have to be selective, thus longer and implying the use of fat suppression pulses. An acceleration factor equal to the one of the SMS-EPI sequence would then be required to match the desired resolutions.

Another advantage of the 3D-EPI sequence also was the 9 dB lower acoustic noise produced by the scanner during the acquisition, thereby facilitating the hearing of auditory stimuli. Nevertheless, the increased acoustic noise level in this case for the SMS-EPI was found to be due to the orientation of the readout gradient (Left-Right for SMS-EPI versus Superior-Inferior for 3D-EPI) and not to the slice selection gradients. Other orientations for the SMS-EPI, e.g. as in [50], thus would lead to a comparable noise level to the one of the 3D-EPI with sagittal orientation.

The signal variance of the fMRI time series originates from both thermal and physiological sources [51]. Their dominance inside a voxel depends on its SNR: for low values, thermal noise is predominant [16]. As the thermal noise is independent of the signal strength, modelling it with signal-dependent regressors (e.g. movement and physiology) does not reduce the variance of the time series. Because the threshold at which this dominance occurs depends on the sequence [9], in agreement with the literature [7, 13, 14] we could show in this study the gain of including the signal-dependent nuisance regressors for the 3D-EPI sequence in terms of d’ even in this moderate tSNR regime, as compared to the SMS-EPI sequence where no improvement was observed. Our pilot study revealed no gain brought by those regressors when based on our physiological recordings (perhaps due to poor quality signals), while the CompCor [47] data-based method systematically improved the results for the 3D-EPI sequence, as expected since this sequence has been shown to be more sensitive to physiological noise. The slightly higher tSNR values for the 3D-EPI can presumably be attributed to a different reconstruction pipeline and smaller g-factor. In addition to the larger matrix size involved in Fourier transform, beneficial for noise averaging [6], the smaller total acceleration of 8 (versus 10) of the 3D-EPI, enabled by a water-selective pulse sparing a fat saturation pulse, can partly explain this difference. Reconstruction details certainly have an impact as well, and slice leakage in the SMS case could affect the results if the leak-block approach was not used [52]. However, it was beyond the scope of this work to tweak reconstruction parameters, as comparing the two sequences with standard reconstruction packages, and their default parametrization, was also one goal of this work. Noise whitening with FAST instead of AR(1) decreased the sensitivity and increased the specificity of the activations for the SMS-EPI sequence which suggests the presence of temporal correlations in the signal that are correctly modelled with FAST [31, 46]. Regarding d’ results, the two sequences showed the same detection performance. The 3D-EPI approach however benefited more from the extent of smoothing than the SMS-EPI in terms of d’. The 5 mm size-smoothing kernel here was kept as a reference to be consistent with the results of the study in [32]. The sensorimotor activations to be observed furthermore are not fine-grained and therefore justify the use of smoothing. The SMS-EPI returned slightly stronger activations or t-scores, especially for high level contrasts. In [52], different MB factors were tried and studied. While the tSNR per square root of time consistently increased from MB factors 1 to 6, the factor of 4 returned stronger activation results, which was consistent with the t-score testing for the mean. In the end, despite the many benchmarking metrics used in this work, we did not find a significant difference between the two sequences. The tSNR and t-score for the mean gain (average values and maps in Fig 4) for the 3D-EPI compared to the SMS-EPI involved mostly central brain regions which are less critical for the task activations.

Limits of this study include the use of reference ROIs for the detection of functional activations: the ROIs obtained in [32] were considered here as ground truth given their robustness and reproducibility across 81 subjects, but yet with remaining small inter-subject variations. The gain in sensitivity and specificity versus temporal resolution also is known to be complex [42, 53] and thus it cannot be claimed that the used protocol parametrizations were optimal for this work. Instead, close to a state of the art setting for resting state fMRI at 7T [35], we attempted to use a robust localizer [32] as an evaluation metric complementary to standard tSNR and t-score. In agreement with [42, 54], our results show that different metrics have their pros and cons and that an evaluation as close as possible to the application can be beneficial. In addition to the t-score testing for the mean signal, the interest of the d’ metric is to summarize in one number the performance of a sequence for a particular application of interest.

## Conclusion

We evaluated the SMS-EPI and 3D-EPI sequences with similar homogenized excitation profiles in a task fMRI study with matched spatial (1.6 mm) and temporal (1200 ms) resolutions at 7T. Complimentary to the other comparisons that can be found in the literature [10–13], the novelty of this work was to use a real life, well-characterized and robust task-based paradigm to compare the two sequences with detection theory. Both sequences showed similar performance in detecting visual, auditory and motor activations. The 3D-EPI sequence however benefited from much lower energy deposition (lower Ernst flip-angle) and slightly lower total acceleration factor (8 versus 10) thanks to the water-selective pulses sparing the need of fat saturation pulses.
